# Correction: Live imaging of excitable axonal microdomains in ankyrin-G-GFP mice

**DOI:** 10.7554/eLife.109210

**Published:** 2025-09-11

**Authors:** Christian Thome, Jan Maximilian Janssen, Seda Karabulut, Claudio Acuna, Elisa D'Este, Stella J Soyka, Konrad Baum, Michael Bock, Nadja Lehmann, Johannes Roos, Nikolas A Stevens, Masashi Hasegawa, Dan A Ganea, Chloé M Benoit, Jan Gründemann, Lia Y Min, Kalynn M Bird, Christian Schultz, Vann Bennett, Paul M Jenkins, Maren Engelhardt

**Keywords:** Mouse

Thome C, Janssen JM, Karabulut S, Acuna C, D'Este E, Soyka SJ, Baum K, Bock M, Lehmann N, Roos J, Stevens NA, Hasegawa M, Ganea DA, Benoit CM, Gründemann J, Min LY, Bird KM, Schultz C, Bennett V, Jenkins PM, Engelhardt M. 2025. Live imaging of excitable axonal microdomains in ankyrin-G-GFP mice. *eLife* **12**:RP87078. doi: 10.7554/eLife.87078.

Published 3 February 2025

After publication, we realized that the text incorrectly described the status of the mouse strain used in these studies. As indicated in the methods, a “neomycin resistance cassette, driven by the phosphoglycerate promoter (PGK-neo) and flanked by flippase recognition target (FRT) sites, was inserted between exon 42 and the 5’ LoxP site. … The FRT-flanked neo cassette was excised by crossing the Ank3-GFP mouse with a mouse expressing flippase recombinase.” We were mistaken that the flippase-mediated removal of the neo cassette had been performed. We have confirmed by PCR that the neo cassette is still present in these mice in the colonies at the University of Michigan, in Europe, and at the Jackson Laboratory.

Since all of the animals used in this study were derived from the colonies at the University of Michigan, and thus all have the neo cassette present, this error neither affects the results nor the conclusions of the article but should be corrected in order to ensure consistency. We regret this inaccuracy and have corrected the manuscript as described below.

We have corrected the text by removing the statement describing removal of the neo cassette. We also added the details of the submission of this strain to the Jackson Laboratory, which also indicates the presence of the neo cassette on the details page for this strain. These changes are underlined below.

Corrected text:

A neomycin resistance cassette, driven by the phosphoglycerate promoter (PGK-neo) and flanked by flippase recognition target (FRT) sites, was inserted between exon 42 and the 5’ LoxP site. The linearized construct was introduced into 129X1/SvJ ES cells by electroporation, and G418-resistant clones were screened using polymerase chain reaction (Figure 1C). One of the primers was anchored outside of the 5’ homology arm to allow the detection of the FLEx cassette within the correct chromosomal location. ES cells selected for the presence of the FLEx cassette using G418 were injected into C57BL/6NHsd blastocysts. A high percentage of chimeric animals were obtained and bred to C57BL/6NHsd mice to produce heterozygous animals. Mutant mice were backcrossed to C57BL6/J mice (Jackson Laboratory) and were compared to C57BL6/J mice as WT controls. These mice have been deposited at the Jackson Laboratory (B6;129S6-Ank3tm1Pmj/J, JAX strain # 038118).

Original text:

A neomycin resistance cassette, driven by the phosphoglycerate promoter (PGK-neo) and flanked by flippase recognition target (FRT) sites, was inserted between exon 42 and the 5’ LoxP site. The linearized construct was introduced into 129X1/SvJ ES cells by electroporation, and G418-resistant clones were screened using polymerase chain reaction (Figure 1C). One of the primers was anchored outside of the 5’ homology arm to allow the detection of the FLEx cassette within the correct chromosomal location. ES cells selected for the presence of the FLEx cassette using G418 were injected into C57BL/6NHsd blastocysts. A high percentage of chimeric animals were obtained and bred to C57BL/6NHsd mice to produce heterozygous animals. The FRT-flanked neo cassette was excised by crossing the Ank3-GFP mouse with a mouse expressing flippase recombinase. Mutant mice were backcrossed to C57BL6/J mice (Jackson Laboratory) and were compared to C57BL6/J mice as WT controls.

